# Diffuse Alveolar Hemorrhage in the Setting of Cytarabine Therapy in a Critically Ill Patient

**DOI:** 10.7759/cureus.19575

**Published:** 2021-11-14

**Authors:** Alexander T Phan, Janie Hu, Mufadda Hasan

**Affiliations:** 1 Internal Medicine, Arrowhead Regional Medical Center, Colton, USA; 2 Internal Medicine, St. George's University School of Medicine, St. George's, GRD; 3 Pulmonary and Critical Care Medicine, Arrowhead Regional Medical Center, Colton, USA

**Keywords:** flexible fiberoptic bronchoscopy (ffb), refractory hypoxemia, acute myeloid leukemia (aml), cytarabine, diffuse alveolar hemorrhage

## Abstract

Diffuse alveolar hemorrhage (DAH) is a potentially life-threatening pulmonary condition characterized by hypoxemia with progression to respiratory failure, rapid onset of dyspnea, and blood loss anemia. While hemoptysis may be present and corroborates the diagnosis, it is absent in about half of the cases, resulting in a diagnostic challenge with variable presenting symptoms. Imaging findings on chest x-ray or computed tomography (CT) scans are also non-specific, often showing diffuse bilateral alveolar opacities. Because DAH is an under-recognized diagnosis, physicians should maintain a degree of clinical suspicion for DAH in patients with unexplained airspace opacities and no signs of an infectious etiology. This is especially important in higher-risk populations such as patients with hematological malignancies, who have a propensity for thrombocytopenia and coagulopathy compounded by the use of anticoagulants. Patients with hematological malignancies, namely acute myeloid leukemia (AML), are also at risk for drug-induced DAH due to the use of cytotoxic medications like cytarabine. Here, we present the case of a 48-year-old male with a past medical history of AML and myeloid sarcoma who developed shortness of breath after receiving cytarabine chemotherapy. Chest radiography revealed diffuse bilateral infiltrates. He was intubated and underwent flexible bronchoscopy, which resulted in a bloody effluent consistent with DAH. After ruling out infectious etiologies, we reached a final diagnosis of DAH and started the patient on corticosteroid therapy.

## Introduction

Diffuse alveolar hemorrhage (DAH) is a potentially life-threatening pulmonary condition characterized by hypoxemia with progression to respiratory failure, rapid onset of dyspnea, and blood loss anemia [1]. While hemoptysis may be present and corroborates the diagnosis, it is absent in about half of the cases, resulting in a diagnostic challenge with variable presenting symptoms [2,3]. Imaging findings on chest x-ray or computed tomography (CT) scans are also non-specific, often showing diffuse bilateral alveolar opacities. Because DAH is an under-recognized diagnosis, physicians should maintain a degree of clinical suspicion for DAH in patients with unexplained airspace opacities [1]. This is especially important in higher-risk populations such as patients with hematological malignancies, who have a propensity for thrombocytopenia, coagulopathy compounded by the use of anticoagulants, and pulmonary infections with *Staphylococcus aureus*, *Pneumocystis jirovecii*, mycobacteria, and cytomegalovirus [3,4]. Patients with hematological malignancies, namely acute myeloid leukemia (AML), are also at risk for drug-induced DAH due to the use of cytotoxic medications like cytarabine [4].

The mechanism of cytarabine-induced damage to the alveolar-capillary membrane is thought to be cytokine-mediated [5]. Although a rare complication, DAH secondary to cytarabine should be considered as a differential diagnosis in AML patients presenting with acute respiratory distress and lung infiltrates of unclear etiology [5]. After ruling out infectious causes or clinical deterioration despite antimicrobial coverage, physicians should consider obtaining a bronchoscopy with bronchoalveolar lavage [1]. In DAH, bronchoscopy would reveal blood on serial lavage.

The general management of DAH includes supportive care with oxygen supplementation or mechanical ventilation, avoiding anticoagulation, and reversing coagulopathy [2]. A short course of systemic steroids is also recommended in addition to rituximab, cyclophosphamide, or plasma exchange depending on the etiology [6]. In patients with suspected drug-induced DAH, it is also important to withhold the offending agent [2].

## Case presentation

A 48-year-old male with a past medical history of alcohol use disorder, myeloid sarcoma, and AML presented to the emergency department complaining of dizziness, fatigue, and fever after receiving cytarabine chemotherapy earlier in the day. He denied chest pain, abdominal pain, nausea, vomiting, headaches, and leg swelling. Of note, five months prior, the patient was diagnosed with AML, which presented as myeloid sarcoma, and he was subsequently started on chemotherapy with idarubicin and cytarabine. The patient’s initial vital signs included a temperature of 99.1 F, pulse rate of 112, respiratory rate of 30, and blood pressure of 101/61 mmHg. Physical examination was notable for pale conjunctiva, tachypnea, slow capillary refill, and tachycardia; the remainder of the examination was unremarkable.

**Figure 1:**
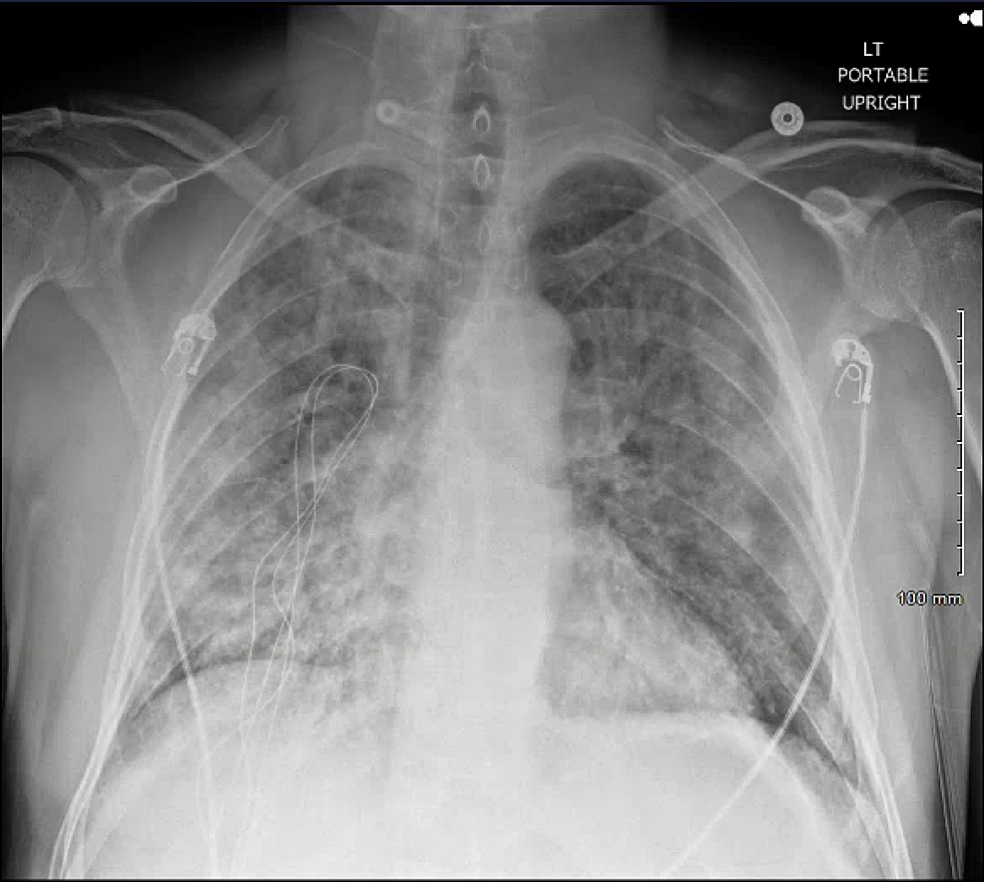
Chest radiograph demonstrating diffuse bilateral opacities.

**Figure 2:**
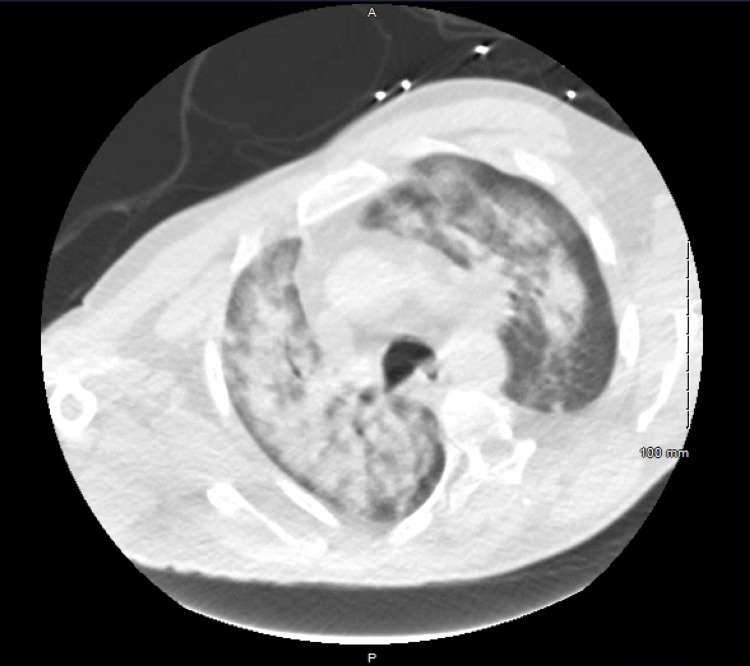
Chest computed tomography with intravenous contrast demonstrating bilateral ground-glass opacities.

The patient's laboratory findings are shown in Table 1, demonstrating leukocytosis with bandemia, severe anemia, severe thrombocytopenia, decreased lymphocytes, elevated metamyelocytes, and elevated myelocytes. The patient was given 1 unit of packed red blood cells for symptomatic anemia and started on vancomycin 1.25 g intravenously (IV), cefepime 1 g IV, and azithromycin 500 mg orally (PO) for empiric broad-spectrum antibiotic coverage due to concerns for sepsis. Shortly after the transfusion, the patient was found to be persistently tachypneic with respiratory rates greater than 30 and had difficulty completing his sentences; consequently, he was started on bilevel positive airway pressure via a face mask. A chest x-ray (Figure 1) and computed tomography (CT) of the chest with intravenous contrast (Figure 2) was obtained and revealed diffuse bilateral, ground-glass opacities, which was concerning for transfusion-related acute lung injury (TRALI) given the recent transfusion. Due to the patient’s increased work of breathing and impending respiratory failure, the patient underwent elective endotracheal intubation and was admitted to the intensive care unit (ICU).

**Table 1:** Significant findings from initial laboratory results. WBC = white blood cells; RBC = red blood cells.

	WBC (cells/μL)	Hemoglobin (g/dL)	RBC (million cells/μL)	Hematocrit (%)	Platelets (cells/μL)	Band neutrophils (%)	Lymphocytes (%)	Metamyelocyte (%)	Myelocyte (%)
Reference values	N=4,300-11,000	N=13-17	N=4.5-5.9	N=41-53	N=120,000-360,000	N=2-5	N=20-48	N=0	N=0
Measured	18,300	4.3	1.36	13.4	9,000	21	10	3	1

Despite halting blood product transfusions for several days, the patient’s respiratory status worsened, evidenced by progressively increasing requirements of fraction of inspired O_2_ levels, positive end-expiratory pressures, and peak inspiratory pressures. The patient’s white blood cell count continued to increase despite broad-spectrum antibiotics and a negative sputum culture. His hemoglobin levels were persistently less than 5, prompting a bronchoscopy with bronchoalveolar lavage, which revealed progressively bloody effluent on serial lavage. As a result, a diagnosis of DAH was made, and the patient was started on a five-day course of methylprednisolone 1000 mg IV. The hematology and oncology specialists reviewed the patient’s case and concurred with the diagnosis of DAH and recommended blood product transfusion as needed to maintain a hemoglobin level of >7 g/dL and a platelet count of >20,000 cells/μL.

During the subsequent days of his ICU course, the patient had difficulty weaning from mechanical ventilation, despite transfusion of blood products and empiric antibiotic coverage. Repeat chest x-rays did not reveal improvement in the patient’s bilateral pulmonary infiltrates. Repeat sputum and blood cultures were negative. The patient was later found to be in asystole on telemetry and, unfortunately, resuscitative measures failed to rescue the patient.

## Discussion

**Figure 3:**
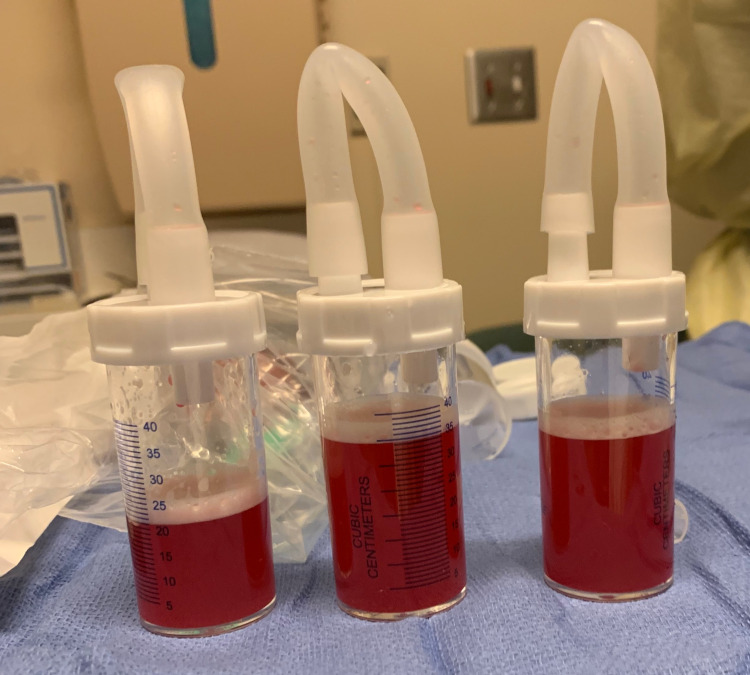
Bloody effluent from bronchoalveolar lavage.

DAH is a life-threatening condition that is most commonly a result of systemic vasculitides; however, it is less commonly associated with cytotoxic drugs and malignancy [1,2]. A diagnosis of DAH in the setting of cytarabine treatment is extremely rare, with only several case reports noted in the current literature [1,3,4,6]. Differential diagnoses for DAH include infectious and non-infectious causes of pulmonary edema such as TRALI, exacerbation of congestive heart failure, bronchiolitis obliterans, acute eosinophilic pneumonia, aspiration pneumonitis, neurogenic pulmonary edema, opioid overdose, and pulmonary infection [1]. It is imperative that the intensivist rules out infectious etiologies and fluid overload states when considering DAH as a diagnosis [2,3]. In our case, we initially considered TRALI as one of the major differential diagnoses given the temporal proximity of the patient’s blood transfusion; however, despite halting blood product transfusions, continuing mechanical ventilation, and ruling out all other sources of bleeding, his hemoglobin levels continued to decrease. Additionally, despite administering empiric broad-spectrum antibiotics and antifungal agents, and negative blood, urine, and sputum cultures, the patient had persistent leukocytosis, fevers, and increasing peak inspiratory pressures on mechanical ventilation. This prompted us to perform a flexible bronchoscopy to assess for a pulmonary source of bleeding. Sequential bronchoalveolar lavages demonstrated increasingly hemorrhagic effluents, and thus the presumed diagnosis was DAH (Figure 3). Consequently, the patient was started on a five-day course of corticosteroids for DAH.

The next diagnostic challenge was determining the etiology of the patient’s DAH. Toxicology reports were negative, albumin levels were within normal limits, and the patient did not have any signs of congestive heart failure on echocardiography, ruling out toxic and volume overload etiologies. Additionally, although the patient was middle-aged, a urinalysis did not demonstrate hematuria, so we did not test for anti-neutrophil cytoplasmic antibodies, anti-glomerular basement membrane antibodies, anti-nuclear antibodies, or anti-double-stranded DNA antibodies. For this reason, we did not initiate rituximab therapy. Our patient had undergone three cycles of chemotherapy that included cytarabine, and based on the preponderance of our diagnostic work-up, we concluded that cytarabine was the cause of our patient’s DAH, as this pharmacologic agent has extensive known pulmonary toxicities.

The primary limitation to our case study is that we did not pursue testing for antibodies associated with vasculitides. Although this limitation exists, it is our contention that our patient’s DAH was most likely a result of cytarabine therapy, as multiple urinalyses failed to demonstrate hematuria, which represents renal involvement, characteristic of vasculitides. Additionally, the patient’s clinical course and diagnostic work-up support the observations of Maini et al., Kanaya & Kondo, and Kopterides et al., which demonstrated that a diagnosis of cytarabine-induced DAH is possible after ruling out all other possible etiologies of pulmonary hemorrhage [1,4,6]. Thus, when a patient presents with diffuse bilateral, ground-glass opacities on chest radiograph, intensivists should consider DAH as a differential diagnosis due to the severe disease burden and many underlying causes of DAH.

## Conclusions

This case helps elucidate the rare situation in which cytarabine treatment can cause DAH in the setting of AML. Intensivists should maintain a high degree of suspicion for DAH when evaluating and treating patients with a history of AML receiving chemotherapy, who present with respiratory distress, unexplained airspace opacities, and no signs of an infectious etiology.
